# Shared and distinct roles of T peripheral helper and T follicular helper cells in human diseases

**DOI:** 10.1038/s41423-020-00529-z

**Published:** 2020-08-31

**Authors:** Hiroyuki Yoshitomi, Hideki Ueno

**Affiliations:** 1 Department of Immunology, Graduate School of Medicine, Kyoto University, Sakyo-ku Kyoto, 606-8501 Japan; 2 Institute for the Advanced Study of Human Biology, Kyoto University, Sakyo-ku Kyoto, 606-8501 Japan; 3 Department of Microbiology, Icahn School of Medicine at Mount Sinai, New York, 10029 NY USA; 4 Global Health and Emerging Pathogens Institute, Icahn School of Medicine at Mount Sinai, New York, 10029 NY USA

**Keywords:** peripheral helper T cells, T follicular helper cells, CXCL13, Autoantibodies, autoimmune diseases, Autoimmunity, Follicular T-helper cells, Mechanisms of disease

## Abstract

The interactions of CD4^+^ T cells and B cells are fundamental for the generation of protective antibody responses, as well as for the development of harmful autoimmune diseases. Recent studies of human tissues and blood samples have established a new subset of CD4^+^ B helper T cells named peripheral helper T (Tph) cells. Unlike T follicular helper (Tfh) cells, which interact with B cells within lymphoid organs, Tph cells provide help to B cells within inflamed tissues. Tph cells share many B helper-associated functions with Tfh cells and induce B cell differentiation toward antibody-producing cells. The differentiation mechanism is also partly shared between Tph and Tfh cells in humans, and both Tfh and Tph cells can be found within the same tissues, including cancer tissues. However, Tph cells display features distinct from those of Tfh cells, such as the expression of chemokine receptors associated with Tph cell localization within inflamed tissues and a low Bcl-6/Blimp1 ratio. Unlike that of Tfh cells, current evidence shows that the target of Tph cells is limited to memory B cells. In this review, we first summarize recent findings on human Tph cells and discuss how Tph and Tfh cells play shared and distinct roles in human diseases.

## Introduction

In the 1980s, CD4^+^ “helper” T cells were initially defined as T cells capable of providing help to B cells.^1^ During the past two decades, T follicular helper (Tfh) cells have been established as a CD4^+^ subset that is specialized to provide help to B cells in the germinal centers of secondary lymphoid organs (SLOs). Tfh cells are critically involved in the pathogenesis of a wide range of diseases, including autoimmune diseases, allergies, infectious diseases, and malignancies.^2–13^ An SLO-like structure can also develop in the periphery at inflammatory sites, termed tertiary lymphoid structures (TLSs), in a variety of diseases, including infection, autoimmune disease, and malignancy.^14^ CD4^+^ T cells within TLSs share many features with Tfh cells, including the expression of CXCL13, IL-21, CD40L, and ICOS, but often lack expression of the chemokine receptor CXCR5.^15^ A series of studies on CD4^+^ T cells in inflamed rheumatoid arthritis (RA) joints demonstrated the presence of CXCL13-producing PD-1^hi^ CXCR5^−^CD4^+^ T cells that were capable of helping B cells within inflamed tissues,^16,17^ and these CD4^+^ T cells were termed peripheral helper T (Tph) cells.^15^ Subsequent studies have revealed both similarities and differences between Tph and Tfh cells in human tissues and peripheral blood. In this review, we first briefly summarize the phenotype and function of the Tph cells present in inflamed human tissues and discuss how Tph and Tfh cells play redundant and distinct roles in various human diseases.

## Tph cells in inflamed RA joints

RA is progressive autoimmune-mediated arthritis accompanied by the presence of autoantibodies such as rheumatoid factor or anti-citrullinated antibody (ACPA).^18^ Tph cells were initially identified during the study of T cells from inflamed RA joints. Manzo et al. reported in 2008 that these CD4^+^ T cells highly produce CXCL13,^16^ the chemokine that binds to CXCR5 and is crucial for the recruitment of B and T cells and formation of lymphoid follicles.^14^ CXCL13-producing CD4^+^ T cells were colocalized with B cells in RA synovial tissues, suggesting in situ interactions.^16^ The CXCL13-producing CD4^+^ T cells in RA joints were memory cells expressing CD69 and CD45RO, 30% of which expressed CCR7.^16^ A majority of CXCL13-producing CD4^+^ T cells lacked the expression of Bcl-6 and CXCR5, unlike Tfh cells present in germinal centers.^16^ Later, Kobayashi et al. revealed that CXCL13-producing CD4^+^ T cells in inflamed RA joints were PD-1^hi^ CXCR5^−^ CD4^+^ TCRαβ cells that did not express Th-related cytokines, including IFN-γ, IL-4, and IL-17.^17^ These observations suggest that CXCL13-producing CD4^+^ T cells represent a T cell population distinct from Th1, Th2, Th17, and Tfh cells. Recently, Rao et al. demonstrated that PD-1^hi^ CXCR5^−^CD4^+^ T cells in inflamed RA joints could provide help to memory B cells as efficiently as PD-1^hi^ Tfh cells. Similar to those of Tfh cells, the B cell helper activities of PD-1^hi^ CXCR5^−^CD4^+^ T cells are dependent on IL-21 and SLAMF5.^15^ However, PD-1^hi^ CXCR5^−^CD4^+^ T cells express CCR2, CCR5, and CX3CR1,^15,19^ chemokine receptors that localize these cells within inflamed tissues enriched with CCL2, CCL5, and CX3CL1. Given their capacity to provide help to B cells and their location in the periphery, PD-1^hi^ CXCR5^−^CD4^+^ T cells were Tph cells.^15^ Whereas both Tfh and Tph cells are found in lymphoid aggregates in RA joints, Tph cells are the dominant PD-1^hi^ cells that interact with B cells outside lymphoid aggregates.^15^

From a functional perspective, Tph cells are defined as CD4^+^ T cells that provide help to B cells in inflamed tissues in autoimmune diseases. However, Tph cells are often defined solely by their phenotype as PD-1^hi^ CXCR5^−^CD4^+^ T cells, which appears to include non-B helpers, which will be discussed later. Further studies on more refined markers of Tph cells for a deeper understanding of their biology are warranted.

## Tph and Tfh cells in inflamed human tissues

While Tph cells are present within inflamed tissues, very few studies have been performed on inflamed human tissues other than those in RA. For example, while PD-1^hi^ CXCR5^−^CD4^+^ T cells were found in inflamed intestinal tissues in Crohn’s disease,^20^ their function and contribution to disease pathogenesis remain unexplored. The reason there have been few studies on tissue Tph cells might be at least in part due to technical difficulties. Tissue samples usually require a tissue dissociation process with collagenases and proteases, which alter the structure of cell surface markers, including CXCR5 (unpublished observations). As no unique markers have been established for Tph cells and the identification of Tph cells in tissues is currently dependent on high expression of PD-1 and no expression of CXCR5, distinguishing Tph cells from Tfh cells can be difficult. Although other chemokine receptors such as CCR2 could be potential markers of Tph cells, the exact chemokine receptors expressed by Tph cells seem to differ according to the disease and conditions. As the expression of the CXCR5 transcript varies according to the stages of Tfh cell differentiation,^21^ transcript analysis can also be suboptimal to distinguish Tph cells from Tfh cells. Accordingly, there have been a number of studies describing PD-1^hi^ CD4^+^ T cells expressing Tfh-associated genes but lacking robust *CXCR5* and *BCL6* expression as Tfh cells in tissues.^22–25^ These cells might indeed be Tph cells or a mixture of Tfh and Tph cells.

## Circulating Tph cells in autoimmune diseases

Instead of analyzing Tph cells in tissues, many studies have analyzed circulating Tph (cTph) cells in the blood, which are defined as PD-1^hi^CXCR5^−^ CD4^+^ T cells, with the aim of assessing the global activity of Tph cells in autoimmune diseases. A similar approach has been commonly used for the analysis of the Tfh cell response, where circulating Tfh (cTfh) cells in the blood are analyzed as a means to assess the global Tfh response.^26^ Studies of cTph cells revealed that their frequency positively correlated with increased autoantibody titers and disease activity in various diseases, providing a rationale and value for the analysis of cTph cells. In RA, cTph cells were increased in seropositive patients (positive for RF or ACPA) but not in seronegative or spondyloarthropathy patients, and the frequency of cTph cells showed a positive correlation with disease activity in seropositive RA patients.^15^ Similarly, cTph cells coexpressing other activation markers, such as CD38 and MHC class II, were increased in patients with systemic lupus erythematosus (SLE).^27–29^ The frequency of cTph cells in SLE is positively correlated with anti-dsDNA titers, disease activity scores such as SLEDAI, and the frequencies of plasma cells/plasmablasts.^27–29^ cTph cells in SLE exhibited B cell helper activities by producing IL-21,^29^ IL-10, and succinate.^30^ Whereas previous studies indicated that these SLE parameters were highly correlated with the frequency of cTfh cells,^31^ other studies indicated an even higher correlation with cTph cells than cTfh cells.^29^

An increase in circulating Tph cells has also been observed in other autoimmune and inflammatory diseases, such as Sjögren’s syndrome,^32^ IgG4-related diseases (IgG4-RDs),^33,34^ systemic sclerosis,^35^ IgA nephropathy^36^ and type I diabetes.^37^ Some correlations were observed between the frequency of circulating Tph cells and disease activity and/or clinical parameters in these diseases. For example, the frequency of cTfh cells is positively correlated with serum IgG4 levels and the number of involved organs in IgG4-RDs^33,34^ and negatively correlated in IgA nephropathy with the estimated glomerular filtration rate.^36^ In children with type 1 diabetes, an increase in cTph cells highly evident in patients who were positive for multiple autoantibodies.^37^ These observations suggest that cTph-associated parameters can be valuable potential biomarker in some diseases. Nonetheless, it is important to note that the mode of actions by which cTph cells contribute to disease pathogenesis might be independent of B helper functions. As PD-1 can be expressed on human CD4^+^ T cells upon TCR activation, it is plausible that the phenotypically defined cTph cells are functionally distinct in different human diseases and might include recently activated cells that lack the capacity to help B cells. For example, CX3CR1^+^ cTph cells in IgG4-RD seem to be cytotoxic cells, as indicated by the high expression of cytotoxic molecules, including GZMA, PRF1, and GPR56.^34^

Similarly, the phenotype, gene profile, and function of cTph cells differs among diseases, most likely as a reflection of differences in the microenvironment of inflamed tissues. Type I IFN plays a key pathogenic role in SLE, and accordingly, IFN-inducible genes are highly enriched in cTph cells from SLE patients.^29^ Furthermore, cTph cells in SLE upregulate Th1 and cytotoxicity-related genes, such as CXCR3,^27,28^ T-bet, and GZMB.^29^ Further studies on cTph cells, particularly in association with inflamed tissues, will increase our understanding of their association with disease pathogenesis.

## Similarities between Tph and Tfh cells

cTph and cTfh cells have been analyzed in parallel in several autoimmune diseases to compare their frequencies, gene profiles, and functions. These studies showed that key features associated with B helper functions were largely shared. Both subsets express IL-21, CXCL13, PD-1, and TIGIT^15,19,37^ and exert B helper activities in a manner largely dependent on IL-21 and SLAMF5.^15^ High expression of Maf is integral for IL-21 expression in both Tph and Tfh cells.^29,38^

**Fig. 1:**
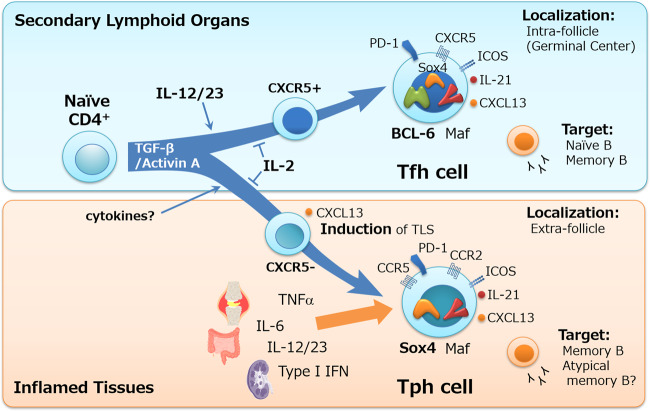
Differentiation of Tfh and Tph cells in humans. Smad2 and Smad3 activation by TGF−β and Activin A is vital for the differentiation of naïve CD4^+^ T cells into Tfh and Tph cells. Environmental IL-2 inhibits the differentiation of both subsets. Activated Stat3 and Stat4 induced by IL-12 and IL-23 stimulation promotes the development of CXCR5^+^ Tfh precursors that migrate toward B cell follicles and eventually differentiate into Bcl-6^hi^ mature Tfh cells. CXCR5^−^ non-Tfh cells exit the secondary lymphoid organs and migrate into peripheral inflamed tissues under the direction of chemokine receptors (CCR2, CCR5, CXCR3, CX3CR1, etc.). TGF−β stimulation contributes to the production of CXCL13 by Tph-like cells via Sox4 and, as such, to the formation of tertiary lymphoid structures (TLSs) in inflamed tissues. Both Bcl-6 and Sox4 likely contribute to CXCL13 expression by Tfh cells. Tph-like cells might gain Th1 and Tfh-like features in inflamed tissues in response to environmental cytokines, including IL-12, IL-23, IL-6, and type I IFN, in the inflamed tissues. Alternatively, Tfh and Tph cells might coevolve in inflamed lymphoid organs in response to Tfh-promoting cytokines, and Tph precursors differentiate into mature Tph cells in the periphery. In TLSs, Tph cells can localize outside lymphoid follicles (extrafollicles) and provide help to memory B cells. CXCR5^−^ atypical memory B cells might also be a target of Tph cells. The transcription factor Maf is integral for IL-21 expression in both Tph and Tfh cells

Notably, the frequencies of cTph and cTfh cells are positively correlated in patients with RA and SLE, as well as in healthy subjects,^15,19,29^ suggesting that Tph and Tfh cells generally codevelop in our body in both healthy and disease states. This is probably consistent with the fact that Tfh and Tph cells share key cytokines for their differentiation in humans: TGF-β and Activin A^39–41^ (Fig. 1). IL-12 and IL-23 act as important partners of TGF-β and Activin A for Tfh cell differentiation.^39,40^ STAT3 and STAT4 are activated by IL-12 and IL-23 and act in concert with Smad2-Smad3, which are activated by TGF-β and Activin A, and induce Tfh molecules, including Bcl-6, IL-21, PD-1, and CXCR5, while downregulating Blimp-1.^39,40^ In contrast, TGF-β alone is sufficient to induce CXCL13-producing Tph-like cells by promoting the expression of the transcription factor Sox4.^38,41^ Sox4 induces CXCL13 expression in human naïve CD4^+^ T cells more strongly than Maf or Tox.^38^ Furthermore, TGF-β and Activin A markedly inhibit the expression of Blimp-1,^39,40^ which suppresses CXCL13 production.^38^ On the other hand, Sox4 does not stimulate human CD4^+^ T cells to express genes associated with B helper functions, such as SH2D1A, IL21, and SLAM family members.^38^ Nonetheless, Tph cells in RA joints^15^ and SLE blood^29^ express Tfh-associated genes, as well as Th1-associated genes, including *TBX21* (T-bet), *IFNG*, *PRDM1* (Blimp-1), *CXCR3*, and *CCR5*. Thus, it is possible that CXCL13-producing Tph-like cells differentiated by Sox4 gain Th1 and Tfh-like features in inflamed tissues in response to environmental cytokines, including IL-12, IL-23, and type I IFN (Fig. 1). Alternatively, Tfh and Tph cells might coevolve in inflamed lymphoid organs in response to Tfh-promoting cytokines, and CXCR5^−^ cells exit and differentiate into mature Tph cells in the periphery. Little is known regarding the plasticity of Tph and Tfh cells, yet it is possible that Tfh cells become Tph cells and vice versa.

IL-2/STAT5 pathway signaling strongly inhibits the differentiation of both human Tfh and Tph cells.^39–41^ Tph cells dominate and initiate the formation of TLSs in RA inflamed joints, where TGF-β is rich but IL-2 is limited.^42,43^

## Differences between Tph and Tfh cells

Other than CXCR5 expression, a major difference between Tph and Tfh cells is the expression of Bcl-6. Bcl-6 is the Tfh-defining transcription factor, which represses transcription factors that are fundamental for the differentiation of other Th linage cells.^44^ Tph cells express lower Bcl-6 and higher Blimp-1 than Tfh cells.^15,29^ As Bcl-6 is highly associated with the localization of Tfh cells in B cell follicles by upregulating CXCR5 and downregulating CCR7, PSGL1, and EBI2,^45^ an increased ratio of Blimp-1 to Bcl-6 likely plays a fundamental role in the peripheral localization of Tph cells, which express the tissue-homing receptors CCR2, CCR5, CXCR3, and CX3CR1.^15,19^

Notably, the ability of Tph cells to help naïve B cells is limited, whereas both subsets can provide efficient help to facilitate memory B cells becoming Ig-producing cells. Naïve B cells produce only IgM upon coculture with autologous cTph cells, whereas naïve B cells cocultured with cTfh cells produce class-switched IgG and IgA.^19^ Similar findings were reported in CXCR5^−^CD4^+^ T cells in the blood of healthy individuals.^2^ Thus, unlike Tfh cells, the target of Tph cells seems to be limited to memory B cells.

The colocalization of Tph cells with B cells can also occur outside TLSs and lymphoid aggregates in inflamed RA joints.^15^ Tph cells may interact with extrafollicular “atypical memory” CD11c^+^CD21^−^ B cells that also lack the expression of CXCR5.^46,47^ Indeed, both CD11c^+^CD21^−^CXCR5^−^ B cells and Tph cells are found in inflamed tissues such as lupus nephritis tissues.^30,48^ Furthermore, the frequency of CD11c^+^CD21^−^CXCR5^−^ B cells is highly associated with Tph cells in SLE blood.^29,30^ Notably, in autoimmune diseases such as SLE, CD11c^+^CD21^−^CXCR5^−^ B cells maintain the capacity to respond to BCR stimulation and differentiate into antibody-producing cells,^46,47^ whereas CD11c^+^CD21^−^CXCR5^−^ B cells in chronic infectious diseases are more prone to becoming anergic or exhausted.^49^ Thus, Tph cells might promote the differentiation of CD11c^+^CD21^−^CXCR5^−^ B cells into autoantibody-producing cells outside TLSs and lymphoid aggregates.

## Tph and Tfh cells in malignancies

T-B cell aggregates and sometimes TLSs can form within and in the area surrounding malignant tumors. Recent studies demonstrated that TLS formation and intratumoral B cell infiltration were associated with improved survival of patients with several types of malignant tumors, such as sarcoma, melanoma, and renal cancer.^50–52^ TLSs in the tumor site can form a mature GC structure containing follicular DCs. Previous studies have shown that B cells in the TLS are clonally expanded, display a switched memory phenotype, and undergo somatic hypermutation.^53,54^ These observations indicate that TLSs at the tumor site are functional and contribute to the activation, expansion, and differentiation of B cells in situ. Previous studies also linked the presence of CXCL13-producing CD4^+^ T cells in malignant tumors with improved survival.^24,55^ Robust CXCL13 production by Tph and Tfh cells^23,24,27,44,52,55^ might contribute to the formation of TLSs within tumors. The precise antitumoral roles of B cells, as well as Tfh and Tph cells, remain to be determined.

## Closing remarks

The features of Tph cells likely differ among diseases by the adoption of signals from microenvironmental factors within the inflamed tissues. Currently, studies on tissue Tph cells are limited, probably due to technical difficulties. It will be important and valuable to assess the features of Tph cells that are specific for each inflammatory disease and tissue and to elucidate their precise functions in inflamed tissues, together with their location and interactions with other cells. Such studies will provide novel insights into disease pathogenesis and novel therapeutic targets.
